# Adipose stromal vascular fraction attenuates T_H_1 cell-mediated pathology in a model of multiple sclerosis

**DOI:** 10.1186/s12974-018-1099-3

**Published:** 2018-03-13

**Authors:** Annie C. Bowles, Rachel M. Wise, Brittany Y. Gerstein, Robert C. Thomas, Roberto Ogelman, Regan C. Manayan, Bruce A. Bunnell

**Affiliations:** 1 Center for Stem Cell Research and Regenerative Medicine, Tulane University School of Medicine, New Orleans, LA USA; 2 Department of Cell and Molecular Biology, Tulane University School of Science and Engineering, New Orleans, LA USA; 3 Neuroscience Program, Tulane University School of Science and Engineering, New Orleans, LA USA; 4 Department of Pharmacology, Tulane University School of Medicine, 1430 Tulane Avenue, SL-99, New Orleans, LA 70112 USA

## Abstract

**Background:**

The therapeutic efficacy of adipose-derived stem cells (ASCs) has been investigated for numerous clinical indications, including autoimmune and neurodegenerative diseases. Less is known using the crude adipose product called stromal vascular fraction (SVF) as therapy, although our previous studies demonstrated greater efficacy at late-stage disease compared to ASCs in the experimental autoimmune encephalomyelitis (EAE) mouse, a model of multiple sclerosis. In this study, SVF cells and ASCs were administered during the pathogenic progression, designated as early disease, to elucidate immunomodulatory mechanisms when high immune cell activities associated with autoimmune signaling occur. These implications are essential for clinical translation when considering timing of administration for cell therapies.

**Methods:**

We investigated the effects of SVF cells and ASCs by analyzing the spleens, peripheral blood, and central nervous system tissues throughout the course of EAE disease following administration of SVF cells, ASCs, or vehicle. In vitro, immunomodulatory potentials of SVF cells and ASCs were measured when exposed to EAE-derived splenocytes.

**Results:**

Interestingly, treatment with SVF cells and ASCs transiently enhanced the severity of disease directly after administration, substantiating this critical immunomodulatory signaling. More importantly, it was only the EAE mice treated with SVF cells that were able to overcome the advancing pathogenesis and showed improvements by the end of the study. The frequency of lesions in spinal cords following SVF treatment correlated with diminished activities of the T helper type 1 cells, known effector cells of this disease. Co-cultures with splenocytes isolated from EAE mice revealed transcripts of interleukin-10 and transforming growth factor-β, known promoters of regulatory T cells, that were greatly expressed in SVF cells compared to ASCs, and expression levels of signaling mediators related to effector T cells were insignificant in both SVF cells and ASCs.

**Conclusion:**

This is the first evidence, to date, to elucidate a mechanism of action of SVF treatment in an inflammatory, autoimmune disease. Our data supports key immunomodulatory signaling between cell therapies and T cells in this T cell-mediated disease. Together, treatment with SVF mediated immunomodulatory effects that diminished effector cell activities, promoted regulatory T cells, and reduced neuroinflammation.

## Background

Adipose is one of the most abundant tissues in the body with many regulatory functions, including immune system regulation. Subcutaneous adipose tissue can be readily and repeatedly collected and contains a high yield of heterogeneous cells once digested. Using a rapid digestion procedure, a heterogeneous combination of cells, including adipocytes, hematopoietic cells, endothelial cells, and various leukocytes that together are known as the stromal vascular fraction (SVF), can be obtained [1–3]. SVF is currently used in both cosmetic and reconstructive clinical procedures, called cell-assisted lipotransfer, when fat grafting is supplemented with SVF cells and re-administered back to the patients where supplementation is desired [3–5]. Many believe that the addition of SVF cells reduces resorption of the fat graft, thus maintaining the volume of the graft. Although this may suggest immunomodulatory effects by SVF cells, the mechanisms have yet to be validated [3, 5]. Current veterinary practice has demonstrated meaningful success with the thousands of procedures using SVF to treat bone, tendon, and soft tissue defects as well as osteoarthritis in horses and dogs without reports adverse effects [6, 7].

Considering the well-documented immunomodulatory effects of adipose-derived stromal/stem cells (ASCs) that reside within the SVF [8–10], SVF cells were investigated for their therapeutic efficacy for an inflammatory, autoimmune diseases such as multiple sclerosis (MS). Using the experimental autoimmune encephalomyelitis (EAE) mouse model of MS, our group has previously reported that treatment with SVF cells at late-stage EAE disease provided robust immunomodulatory effects to peripheral lymphoid organs, anti-inflammatory effects in CNS tissues, and partial restoration of motor function within 10 days. The induction of regulatory T cells (Tregs), high levels of interleukin-10 (IL-10), and polarization of macrophages to an alternative activation, or M2, phenotype were attributed to the comprehensive improvements [11].

In this study, using SVF cells and ASCs during early EAE disease when the pathogenesis is rapidly progressing due to the high inflammatory and autoimmune activities was investigated. T helper type 1 (T_H_1) cells are main effector cells mediating many autoimmune and inflammatory diseases including MS and EAE [12]; therefore, we interrogated the T cells’ subtypes for immunomodulatory effects. SVF cells, ASCs, and vehicle were administered at 8 days post induction (DPI). Spleens, peripheral blood, and CNS tissues from EAE mice were analyzed 6 days after treatment which showed the early effects following treatment. The suppression of pro-inflammatory factors in the sera and the spleen indicated interleukin-2 (IL-2)-dependent abrogation of T_H_1 cell differentiation and associated functions with SVF treatment. This data suggests that SVF treatment attenuated differentiation of T_H_1 cells by disrupting the synthesis of transcription factor STAT1 which led to decreased T-bet, the master regulator of T_H_1 cells. Concomitantly, high levels of IL-10 and transforming growth factor-β (TGFβ) were induced which evidenced the start of Treg differentiation. Although treatment with ASCs also reduced IL-2 signaling, the subsequent high levels of interleukin-6 (IL-6) suggests confounding effects in the CNS tissues following treatment with ASCs. CNS pathology showed modest differences amongst all groups; however, the results of SVF administration suggest that the immunomodulation in the periphery attenuated CNS pathology and reduced the severity of disease.

This evidence demonstrated therapeutic efficacy of SVF cells during active autoimmunity and inflammatory activities. The mechanisms used by SVF cells and ASCs to counter the inflammatory autoimmune activities in vitro were identified. We demonstrated that the repertoire of the T cells contained amongst the cells within the SVF is mainly of T_H_2 cells and, when exposed to the pathologic environment, SVF rapidly increased the levels of IL-10 and TGFβ. Once administered to EAE mice, SVF attenuated the activities of T_H_ cell subsets which reduced the pathological progression in the CNS tissues. This is the first evidence, to date, that revealed a mechanism that directly suppressed the activities of T_H_1 cells by markedly reducing the gene expression of transcription factors responsible for the differentiation of T_H_1 cell. These potential mechanisms shed light on the mechanisms that result in beneficial effects such as volume retention during the cell-assisted liposuction for fat grafting and other current practices following the use of SVF. Regardless of the mechanistic effects that lead to immunomodulation, the use of SVF cells and ASCs should be used with caution during active disease due to the highly proliferative states of the pathogenic T cells. Due to this and the evidence from our study, the timing of the administration is critical, especially during advancing pathogenesis like during relapse phases of MS, when cell therapies could potentially have detrimental effects.

## Methods

### EAE induction using myelin oligodendrocyte glycoprotein_35–55_ peptide

Reagents for EAE induction were prepared by dilution of myelin oligodendrocyte glycoprotein_35–55_ peptide (MOG; 2 mg/mL; AnaSpec, Fremont, CA) in UltraPure™ distilled water (Invitrogen, Life Technologies, Grand Island, NY) emulsified with equal parts of complete Freund’s adjuvant (BD Biosciences Franklin Lakes, NJ) containing 8 mg/mL *Mycobacterium tuberculosis* H37RA (#231131; BD Biosciences). MOG emulsion was transferred to 1 mL Luer-Lok syringes (BD Falcon) with 27G ½” needles. Pertussis toxin was diluted in UltraPure™ (2 ng/μL; List Biologicals Laboratories, Campbell, CA) and transferred to syringes as described above. While anesthetized under 5% isoflurane gas, 6- to 8-week-old female C57Bl/6 mice (Charles River Laboratories, Wilmington, MA) were induced with EAE by subcutaneous injection of 100 μL MOG emulsion near flanking regions of the tail (200 μL total per mouse). Mice were concomitantly administered 100 μL pertussis toxin via intraperitoneal (IP) injection. After 48 h, mice received a second IP injection of 100 μL pertussis toxin to complete the induction procedure. Mice designated in the sham control group received equivalent volumes and injections containing only Hank’s balanced salt solution (HBSS). All animal procedures were approved by the Institutional Animal Care and Use Committee at Tulane University and were in compliance with state and federal National Institute of Health’s animal welfare regulations. Mice were given food pellets and water ad libitum.

Using a standard clinical scoring system, mice were scored by blinded researchers daily for each group (*n* = 5 mice) starting the day of EAE induction 0 days post-induction (DPI) to 30 DPI. Mice were scored based on a standard scoring system. Briefly, a score of 0 is no observable signs of disease, 1 is limp tail with abnormal gait, 2 is hind limb weakness, 3 is partial paralysis of the hind limbs, 4 is complete paralysis of hind limbs, and 5 is moribund or dead. Every 5 days, each mouse was placed in a cylindrical arena and video recorded for a 5-min duration, as previously described [13]. Only mice induced with EAE were randomly designated to the treatment groups. During the 5 min, rearing behaviors were assessed for mice in each group (*n* = 5) by blinded researchers.

### Isolation of the SVF cells and ASCs

Inguinal white adipose tissue was collected from enhanced green fluorescence protein (GFP) transgenic female mice (C57Bl/6-Tg(UBC-GFP)30Scha/J strain; Jackson Laboratory, Bar Harbor, ME) between the ages of 6–12 weeks. Adipose was washed 3–4 times with phosphate buffered saline (PBS; Hyclone Laboratories, Inc., Logan, UT) and finely minced. Adipose was then transferred to a digestion solution containing 0.1% (*w*/*v*) collagenase type I (Sigma-Aldrich, St. Louis, MO) and 1% (*w*/*v*) bovine serum albumin (Sigma-Aldrich) in PBS and placed in an incubator shaker set to 100 rpm at 37 °C for 1 h. Digestion reaction was neutralized with complete culture media (CCM) that contained Dulbecco’s modified Eagle medium: Nutrient Mixture F-12 (Life Technologies), 10% fetal bovine serum (Atlanta Biologicals, Inc., Flowery Branch, GA), 100 units/ml antimycotic/antibiotic (Life Technologies), and 2 mM ʟ-glutamine (Life Technologies). Digested tissue was filtered through sterile gauze and centrifuged to obtain the pellet of SVF cells.

For the isolation of ASCs, SVF cells were plated in 15-cm culture dishes containing CCM, which was incubated at 37 °C with 5% humidified CO_2_. After 24 h, plates were washed with PBS to remove non-adherent cells, and fresh CCM was added to each plate. When 70% confluence was achieved, cells were passaged by trypsinization with 0.25% trypsin/1 mM EDTA (Gibco), neutralized with *v*/*v* CCM, centrifuged, and re-seeded onto culture dishes at a density of 100–200 cells/cm^2^. This process was repeated for subsequent passages; then, cells were cryopreserved prior to experimental use.

### Preparation and injection of cells

Based on past EAE studies, the day for early-stage treatment, which coincides with the start of the inflammatory phase of EAE, was designated as 8 DPI. At this time, the initial signs of motor impairment were observed. At 8 DPI, PBS was used to wash freshly isolated SVF cells and cryopreserved ASC (passage 3–4) prior to injection. Live cells were counted under fluorescence microscopy using acridine orange/ethidium bromide staining method on a hemocytometer [14]. Subsequently, ASCs and SVF cells were re-suspended in Hank’s balanced salt solution (HBSS) at concentrations of 1 × 10^4^ cells/μL and transferred to 1 mL Leur-Lok syringes with 27G ½” needles. EAE mice were randomly designated to treatment groups: ASC treatment, SVF treatment, or vehicle control groups, and received an IP injection of 100 μL containing 10^6^ ASCs, 10^6^ SVF cells, or HBSS, respectively.

### Tissue harvest and processing

**Fig. 1:**
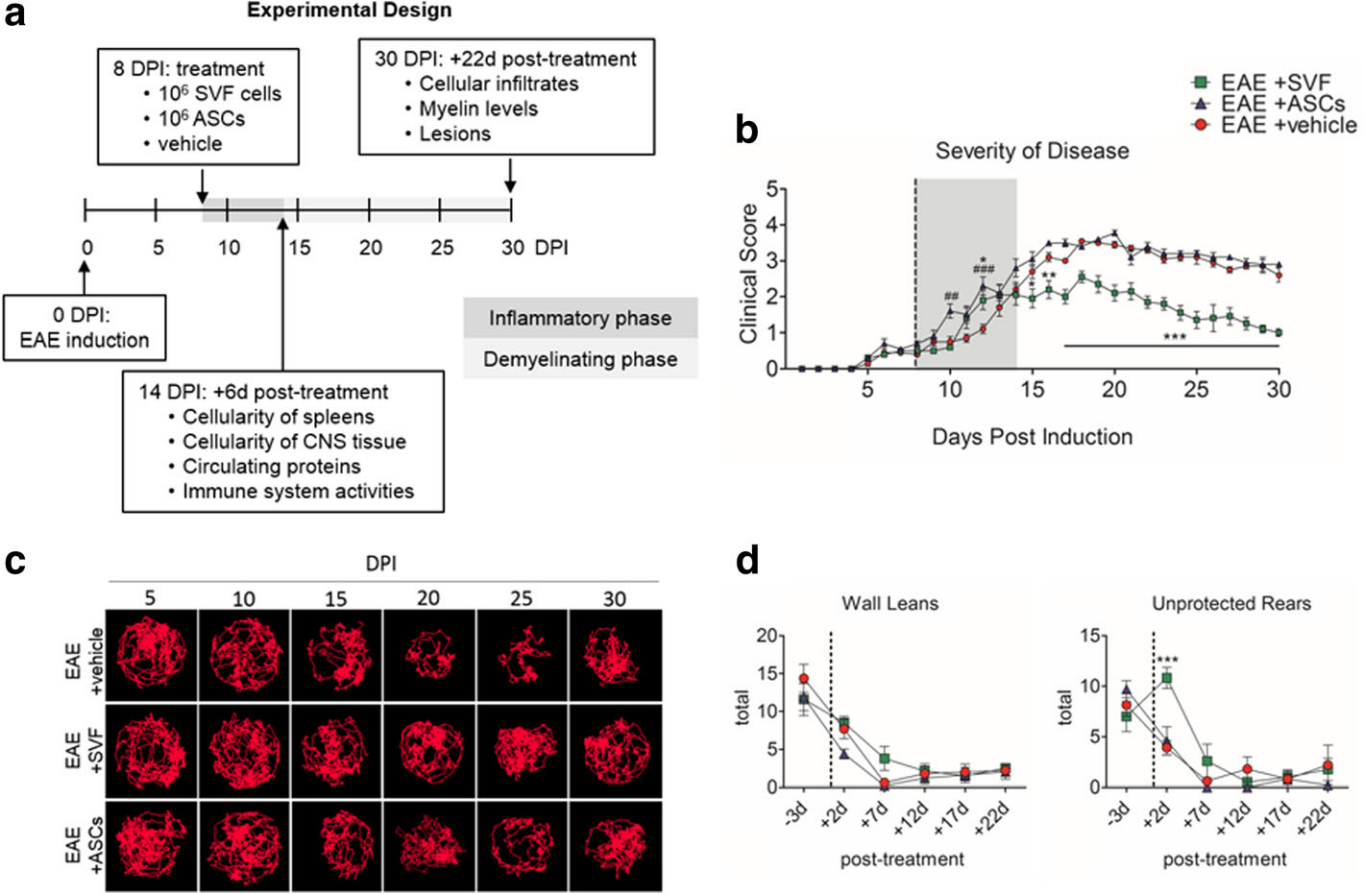
The disease pathogenesis of EAE following treatment at early-stage EAE. **a** Schematic of the experimental design showing the timeline that indicates the main time points for the study. **b** Severity of disease progression for each group throughout the trial. **c** Top down visuals of compiled tracks recorded over a 5-min duration of a representative mouse from each group. **d** Behavioral assessments for each group. An *n* = 5 mice for each group was used for quantitative comparisons, and values were reported as the mean ± SEM. *P < 0.05; *P < 0.01; ***P < 0.001 for EAE + SVF compared to EAE + vehicle; ^#^*P* < 0.05; ^##^*P* < 0.01; ^###^*P* < 0.001 for EAE + ASCs compared to EAE + vehicle

According to the experimental design (Fig. 1a), tissues were harvested for various analyses at 6 days (14 DPI) and 22 days (30 DPI) after treatment. At these time points, EAE mice were euthanized by CO_2_ asphyxiation and blood was immediately collected by intracardial puncture. Following, mice were perfused with sterile PBS, and the spleens and CNS tissues of each EAE mouse were harvested. The blood was allowed to clot at room temperature for 30 min, centrifuged, and then the sera was transferred to tubes and stored at − 80 °C until further use. Cervical sections of spinal cords were removed and stored at room temperature in formalin for paraffin-embedding. Remaining CNS tissues were mechanically digested using a 15-mL Dounce homogenizer. The homogenates were then passed through a cell strainer into a 50-mL conical tube and centrifuged at 2000 rpm for 5 min. Ficoll-Paque™ Premium (GE Healthcare Bio-Sciences, Pittsburgh, PA) was prepared fresh as the 100% isotonic solution by diluting nine parts Ficoll-Paque™ Premium with one part × 10 PBS. Additional solutions (40 and 70%) were prepared by dilution with HBSS. Cell pellets were obtained and re-suspended in 40% isotonic solution that was carefully overlaid on top of 70% isotonic solution in a conical tube. The samples were centrifuged for 30 min at 2000 rpm at 18 °C without the brake. Mononuclear cells from CNS tissues were collected from the 40%/70% interface, washed with PBS, and counted. Samples were then processed for flow cytometric analysis or stored at − 80 °C for RNA isolation.

Harvested spleens were mechanically digested through cell strainers using the blunt ends of syringes. The cells were then incubated with red blood cell lysis for 5 min at room temperature. Following, the splenocytes were washed with PBS and counted. Splenocytes were then processed for flow cytometric analysis or stored at − 80 °C for RNA isolation.

### Flow cytometric staining and analysis

CNS cells and splenocytes were counted and prepared in staining buffer containing 1% bovine serum albumin (BSA; Sigma-Aldrich) and 1% CD16/CD32 Mouse BD Fc Block™ (BD Biosciences). Next, cells were stained with fluorescently conjugated anti-mouse antibodies against CD3, CD4, CD8, CD19, CD25, CD45, CD11b, CD86, and CD206 (eBioscience). Designated samples were incubated in a fixation/permeabilization solution (eBioscience), stained with a fluorescently conjugated anti-mouse antibody against intracellular foxp3, washed, and stored in buffering solution. All other samples were washed and fixed using 1% paraformaldehyde. The samples were stored in at 4 °C until further analysis.

Flow cytometric analysis of each sample was performed using a BD LSRFortessa™ (BD Biosciences) instrument. Data analysis was acquired of 10,000 events using FlowJo software (FlowJo, LLC, Ashland, OR) for representative samples in each group (*n* = 4).

### RNA isolation from spleens

Briefly, Qiazol Tissue Protectant (Qiagen, Valencia, CA) was added to cells isolated from the CNS tissues and spleens. Total RNA extraction was performed for each sample using the RNeasy Mini Kit (Qiagen) according to the manufacturer’s instructions. RNA was subsequently purified with DNase I (Invitrogen) and 1 μg RNA was reverse transcribed using the SuperScript VILO cDNA synthesis kit (Invitrogen). EXPRESS SYBR GreenER qPCR SuperMix Kit (Invitrogen) was used for quantitative reverse transcriptase polymerase chain reaction (qPCR) using mouse-specific primers listed in Table 1. All other primer sequences were previously reported [11]. For GFP detection, qPCR data was calculated and reported as the log (−∆Ct) to represent the levels of GFP mRNA. All other qPCR data was calculated and reported as the ∆∆Ct values that were normalized to the vehicle control group for quantitative comparison of mRNA expression levels. Values were represented as the mean ± SEM of the relative fold change for each group (*n* = 3 mice).

**Table 1:** Primer sequences for mouse-specific transcripts

Transcript	Forward sequence (5’-3’)	Reverse sequence (5’-3’)
Interleukin-2 receptor β	GGCTCTTCTTGGAGATGCTG	GCCAGAAAAACAACCAAGGA
Interleukin-23	CAAAGGATCCGCCAAGGTCT	GGAGGTGTGAAGTTGCTCCA
Foxp3	TTGGCCAGCGCCATCTT	TGCCTCCTCCAGAGAGAAGTG
STAT1	CTTATTCCATGGA CAAGGTTTTG	GGTGCTTCTTAATGAGCTCTAGG
T-bet	AACCAGTATCCTGTTCCCAGC	TGTCGCCACTGGAAGGATAG
GATA-3	CTCCTTTTTGCTCTCCTTTTC	AAGAGATGAGGACTGGAGTG

### Histological analysis of spinal cords

Formalin fixed cervical spinal cords were paraffin-embedded, cut 5 μm thick, and mounted on microscope slides. Sections were stained with hematoxylin and eosin (H&E; Richard-Allan Scientific, Thermo Fisher Scientific) and scanned into Imagescope software (Aperio Technologies, Inc., Vista, CA) using an Aperio Scanscope® CS2 instrument (Aperio Technologies, Inc.). For each section, 3 images of randomly selected fields of white matter were taken at × 40 magnification. Each image was then uploaded onto Fiji/Image J software (National Institutes of Health, Bethesda, Maryland), and cells were counted according to the number of pixels and circularity. For each mouse section, 3 fields were measured for cellular infiltrates per group (*n* = 5).

Luxol fast blue (LFB; IHC World, LLC, Woodstock, MD) staining of spinal cord sections was analyzed using pixel-based algorithms by Imagescope software. For each group, 2 sections for each mouse and 5 mice per group were analyzed to quantify the levels of myelin. Myelin levels were calculated as the percent positive pixels/total number of pixels × 100, and al histological measurements were represented as the mean ± SEM.

### Detection of serum proteins using an enzyme-linked immunosorbent assay (ELISA)

Sera stored at − 80 °C were thawed and equal amounts were pooled together from 7 mice to represent samples for each group. Samples were added to the wells of an ELISArray plate of the Mouse Th1/Th2/Th17 Cytokines Multi-Analyte ELISArray Kit (MEM-003A; SABiosciences, Qiagen) and processed according to the manufacturer’s protocol. The plate was read at absorbance (450 nm), and values were corrected by subtraction of the negative control values.

### SVF cells or ASCs co-cultured with splenocytes isolated at 8 DPI

Spleens were harvest from EAE mice 8 days after disease induction, and the splenocytes were isolated by mechanical digestion as described above. Fresh SVF cells were also isolated as aforementioned; meanwhile, cryopreserved ASCs were thawed and washed. All cells were then counted. Using a transwell® culture system with 0.4 μM pore insert (Corning, Inc., Corning, NY), 500,000 ASCs or SVF cells in culture media were placed into the wells of a 6-well plate in triplicate. Next, the inserts were placed in the wells, and 500,000 splenocytes in culture media were placed in the above chamber. The co-culture system was incubated at 37 °C with 5% CO_2_. After 4 days, the SVF cells and ASCs were collected and pooled together. RNA was isolated, cDNA was synthesized, and gene expression levels of specific primers were analyzed by qPCR as described above. The values were reported as the log(-Ct).

### Statistical analysis

All values reported for the SVF-treated and ASC-treated EAE mice and sham control groups were quantitatively compared to the vehicle-treated EAE group. Statistical analyses were performed using one-way analysis of variance (ANOVA) followed by pairwise comparisons of the mouse groups using Bonferroni post hoc testing. Significance for the overall group effect and individual pairwise comparisons were made against the vehicle-treated EAE group and defined as *P <* 0.05. Analysis was performed using Prism 5.0 (Graphpad Software, La Jolla, CA).

## Results

### Motor impairment was reduced with SVF treatment but not ASCs

EAE mice were treated at the start of the inflammatory phase of disease (8 DPI), designated as early-stage therapy. At 8 DPI, EAE mice treated with vehicle, SVF cells, and ASCs showed comparable disease severity with scores of 0.4 ± 0.1, 0.5 ± 0.0, and 0.7 ± 0.1, respectively. Interestingly at 10 DPI, the ASC-treated EAE had an increased severity (1.6 ± 1.2; *P* < 0.01) compared to the vehicle-treated (0.8 ± 0.1) and SVF-treated (0.6 ± 0.1) EAE groups. Similarly, both ASC-treated (2.3 ± 0.2; *P* < 0.001) and SVF-treated (1.9 ± 0.3; *P* < 0.05) EAE groups were greater in disease severity than the vehicle-treated EAE group (1.1 ± 0.1) at 12 DPI. However, a week after treatment (15 DPI), a marked reduction in disease severity was revealed in the SVF-treated EAE mice (2.0 ± 0.3; *P* < 0.05) compared to the vehicle-treated (2.7 ± 0.2) and ASC-treated (3.1 ± 0.2). Thereafter, SVF-treated EAE continued to show significant reductions in disease severity compared to vehicle-treated EAE mice. EAE mice in both the vehicle and ASC treatment groups were indistinguishable. By the end of the trial (30 DPI), the SVF-treated mice displayed loss of tail tone, mildly abnormal gate, and average score of 1.0 ± 0.1 (*P* < 0.001), whereas the vehicle-treated (2.6 ± 0.2) and ASC-treated (2.9 ± 0.1) remained with severe hind limb weakness (Fig. 1b).

The combined track visuals display the corresponding trend for the severity of disease for each group. The SVF treatment group consistently showed the most tracked distance that was uniformly traced the circular arena. Contrastingly, the ASC and vehicle EAE groups showed a reduction in tracked movement starting at 15 DPI and an unequal distribution of traced area which indicates cognitive impairment (Fig. 1c).

Behavioral parameters require hind limb strength, balance, and coordination which are affected in EAE mice. Mice were assessed 3 days prior to treatment (5 DPI) for wall leans and showed no statistical difference between the vehicle (14.4 ± 1.9), SVF (11.6 ± 2.1), and ASC (11.9 ± 1.8) treatment groups in execution of wall leans, while in the testing arena for a 5-min duration. At 2 days following treatment, EAE mice treated with vehicle, SVF cells, and ASCs were able to perform 7.7 ± 1.3, 8.5 ± 0.8, and 4.4 ± 0.6 wall leans, respectively. A week following treatment (15 DPI), SVF-treated EAE mice were able to perform 3.8 ± 1.6 wall leans, whereas not all of the EAE mice in the vehicle (0.6 ± 0.4) and ASC (0.2 ± 0.2) treatment groups were able to execute a wall lean. At 20 DPI, EAE mice treated with vehicle, SVF cells, and ASCs were comparable with 1.8 ± 1.0, 2.1 ± 1.0, and 1.2 ± 0.8 wall leans, respectively, and remained consistently similar thereafter (Fig. 1d).

Unlike wall leans in which mice are supported by the walls of the testing arena, the ability of mice to execute the unprotected rearing behavior may be more difficult with hind limb impairment. EAE mice designated to the vehicle (8.1 ± 1.1), SVF (7.0 ± 1.5), and ASC (9.7 ± 0.8) treatment groups were able to perform comparable numbers of unprotected rears 3 days prior to treatment. At 2 days following treatment, the SVF-treated mice (10.8 ± 1.0; *P* < 0.001) were able to complete significantly more rears than the vehicle-treated (3.9 ± 0.7) EAE mice. At this time, ASC-treated (4.6 ± 1.4) EAE mice were comparable to the vehicle treatment group and were unable to complete rearing behaviors at 15 and 20 DPI. The SVF-treated EAE mice were able to execute 2.6 ± 1.7 rears while vehicle-treated EAE (0.6 ± 0.4) mice were unable to complete any rears at 15 DPI. At 20–30 DPI, EAE mice in each group performed comparable numbers of rears (Fig. 1d).

### Frequencies of immune cells were enhanced in the CNS tissues in response to treatment with SVF cells and ASCs

**Fig. 2:**
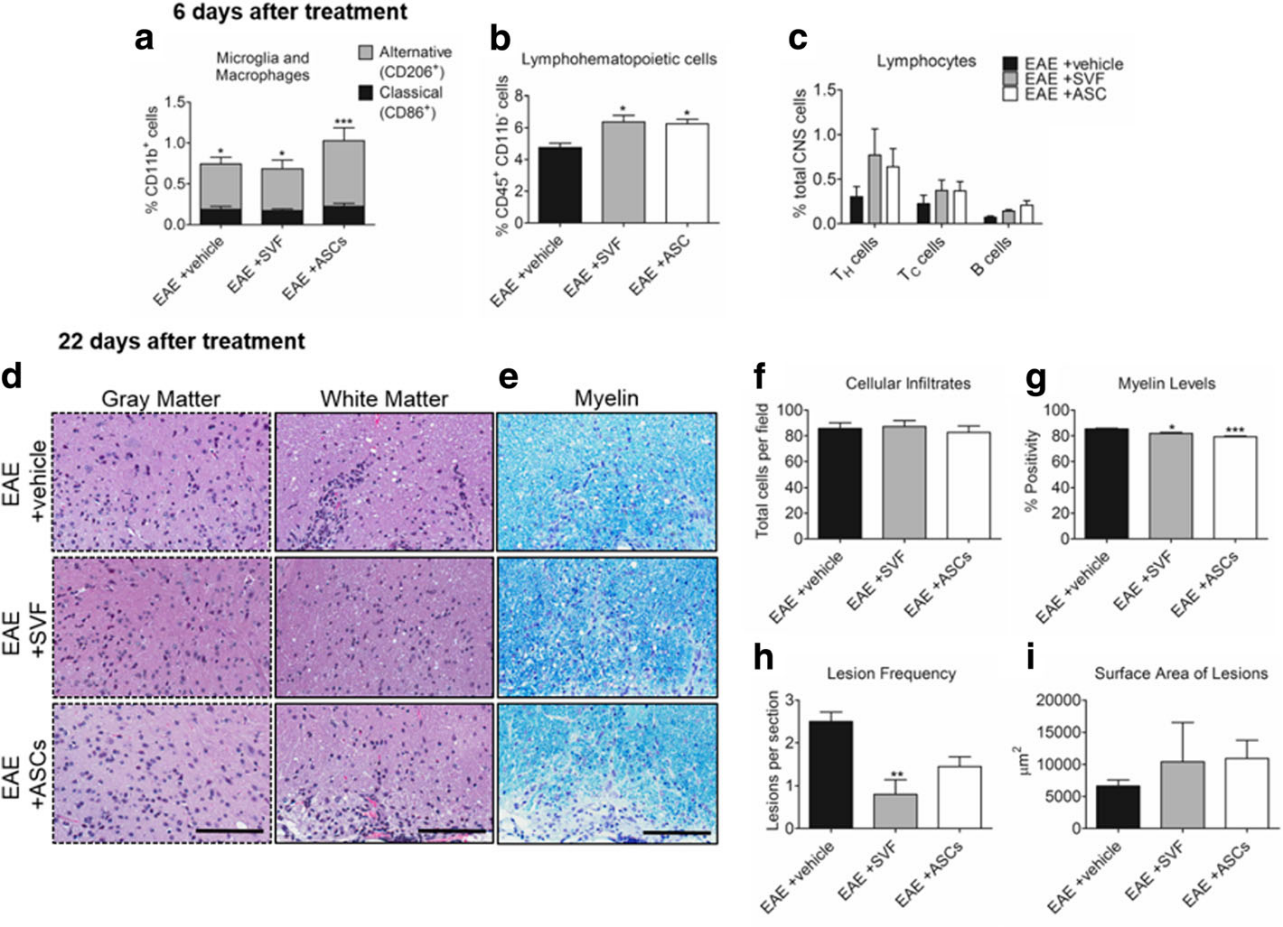
Histological analyses 6 and 22 days following treatment. **a**–**c** Frequencies of cell populations in the CNS of EAE mice 6 days after treatment with SVF cells, ASCs, or vehicle. **d** Images of H&E-stained spinal cord sections used to detect cellular infiltrates. **e** LFB-stained spinal cord sections used to quantify levels of myelin. **f**–**i** Quantitative comparison of histological assessments for each group 22 days after treatment. For each group, tissues from 5 mice were analyzed. Data represented as the mean ± SEM. Scale bar represents 100 μm. *P < 0.05; *P < 0.01; ***P < 0.001

Flow cytometric analysis revealed changes to cell populations detected in the CNS tissues of EAE mice 6 days following treatment at early-stage disease. CD11b^+^CD86^+^ classical activation macrophages were detected at similar frequencies in the vehicle-treated (0.19 *±* 0.04%), SVF-treated (0.17 ± 0.02%), and ASC-treated (0.22 ± 0.04%) EAE mice. In contrast, the frequencies of CD11b^+^CD206^+^ alternative activation macrophages were markedly higher than the classical phenotype in the CNS of vehicle-treated (0.56 ± 0.08%; *P* < 0.05), SVF-treated (0.51 ± 0.12%; P < 0.05), and more significantly the ASC-treated (0.80 ± 0.16%; *P* < 0.001) EAE mice (Fig. 2a).

The frequencies of lymphohematopoietic cells, detected by CD45^+^CD11b^−^ cells, showed marked increases in the CNS 6 days following treatment with SVF cells (6.37 ± 0.41%; *P* < 0.05) and ASCs (6.26 ± 0.29%; *P* < 0.05) compared to the vehicle treatment group (4.76 ± 0.27%; Fig. 2b). As for the mature lymphocyte populations in the CNS, similar trends were detected in the CD3^+^CD4^+^ T_H_ cells, CD3^+^CD8^+^ T_C_ cells, and CD45^+^CD19^+^ B cells following treatments; however, there were modest differences amongst the groups. The frequency of T_H_ cells in the CNS 6 days after SVF treatment (0.77 ± 0.29%) was highest, followed by ASC treatment (0.64 ± 0.20%), and the vehicle treatment (0.30 ± 0.11%) had the lowest frequency. Similarly, T_C_ cells were modestly increased in the SVF-treated (0.37 ± 0.12%) and ASC-treated (0.37 ± 0.11%) compared to the vehicle-treated (0.23 ± 0.10%) EAE group. Although at small frequencies, the detection of B cells showed that ASC treatment (0.21 ± 0.05%) induced the highest increase in frequency followed by SVF (0.14 ± 0.02%) and vehicle (0.07 ± 0.1%) treatments (Fig. 2c).

### CNS histology reveals reduced lesions yet enhanced demyelination 22 days after treatment SVF cells and more so ASCs

The infiltration of cells within the spinal cords of EAE mice were comparable 22 days after treatment with vehicle (85.70 ± 4.50%), SVF cells (87.17 ± 4.76%), and ASCs (82.67 ± 5.06%). Images showed cellular infiltrates that were disseminated throughout the gray and white matter following SVF treatment. On the contrary, treatment with ASCs and vehicle revealed cellular infiltrates predominantly localized to the white matter (Fig. 2d, f).

LFB staining permits the detection of myelin and subsequent calculation of myelin levels of spinal cord sections from EAE mice prepared 22 days after treatment (30 DPI). Myelin levels were most reduced following treatment with ASCs (79.27 ± 0.70%; *P* < 0.001) followed by SVF cells (81.85 ± 0.97%; *P* < 0.05) compared to the vehicle treatment group (85.40 ± 0.56%). These levels of myelin are inferential to the level of demyelination influenced by treatment (Fig. 2e, g).

The frequency and surface area of lesions were measured in spinal cord sections from each group. Although the SVF-treated EAE had the most reduced lesion frequency, the average surface area of these lesions were highest (10,405.5 ± 6112.9 μm^2^) compared to the ASC-treated (9126.6 ± 3588.1 μm^2^) and vehicle-treated (4840.1 ± 1149.1 μm^2^) EAE mice (Fig. 2h, i).

### SVF cells co-cultured with splenocytes from 8DPI EAE mice demonstrated rapid anti-inflammatory cytokine production compared to ASCs

**Fig. 3:**
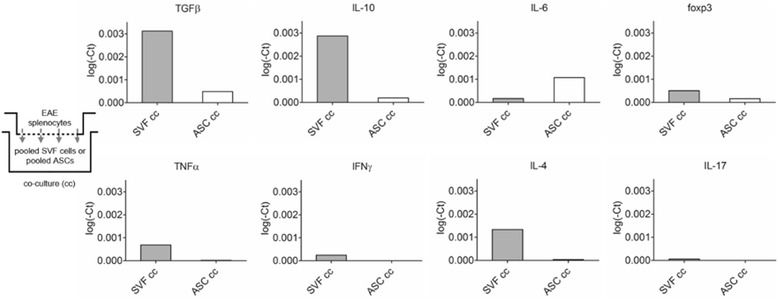
Splenocytes isolated from EAE at 8 DPI were co-cultured 1:1 with SVF cells or ASCs. The SVFs or ASCs were collected and pooled for analysis of gene expression after 4 days of co-culture. Analysis of T cell associated cytokines demonstrated how the administered cells respond to the disease milieu to provide expected outcomes in vitro

The responses from SVF cells and ASCs generated from exposure to the EAE diseased milieu were imitated in a co-culture (cc) system with splenocytes from EAE mice. The gene expressions of relevant cytokines that modulate T cell differentiation or are produced by ASCs or the cells within the SVF directly were analyzed after 4 days. The levels of TGFβ were sixfold higher in SVF co-culture (SVF cc; 3.12 × 10^−3^ log(-Ct)) compared to the ASC co-culture (ASC cc; 4.9 × 10^−4^ log(-Ct)). Similarly, the levels of IL-10 were higher in the SVF cc (2.87 × 10^−3^ log(-Ct)) compared to the ASC cc (1.94 × 10^− 4^ log(Ct)). The levels of IL-6 were measured highest in the ASC cc (1.07 × 10^−3^ log(-Ct)) compared to the SVF cc (1.71 × 10^−4^ log(-Ct)). The regulatory gene *foxp3* is expressed by Tregs as well as mesenchymal stem cells [15]. The expression levels of foxp3 were threefold higher in the SVF cc (5.09 × 10^−4^ log(-Ct)) than the ASC cc (1.66 × 10^−4^ log(-Ct); Fig. 3).

Due to the minimal expression of T cell-related cytokines IL-4, IL-17, IFNγ, and TNFα from the ASCs, these gene expression levels may be attributed to the T cells contained within the heterogeneous SVF cells. Of these cytokines, the levels of IL-4 (1.33 × 10^−3^ log(-Ct)) were highest in the SVF cc compared to IL-17 (5.8 × 10^−5^ log(-Ct)), IFNγ (2.47 × 10^−4^ log(-Ct)) and TNFα (6.91 × 10^−4^ log(-Ct)), respectively. For the ASC cc, the levels of IL-4 (3.77 × 10^−5^ log(Ct)), IL-17 (1.0 × 10^−6^ log(-Ct)), IFNγ (2.00 × 10^−6^ log(-Ct)), and TNFα (1.80 × 10^−5^ log(-Ct)) were negligible (Fig. 3).

### Serum proteins indicate trophic cytokines for T cells that promote proliferation and expansion of specific subsets

**Fig. 4:**
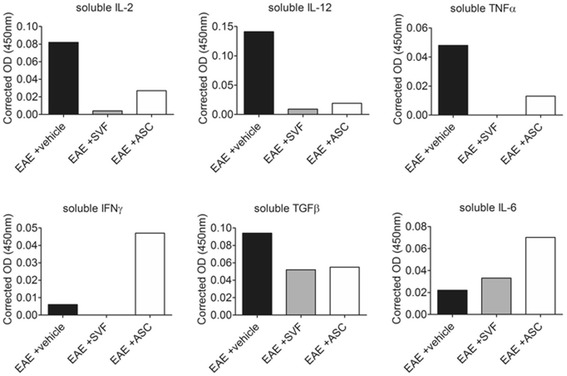
Serum proteins detected 6 days after treatment showed changes to cytokines secreted into the peripheral blood that led to paracrine effects

Soluble cytokines in the peripheral blood circulation of EAE mice that were relevant to the differentiation of or produced by specific T_H_ cells subsets were detected. The levels of IL-2 were highest in the vehicle treatment group (0.082 OD) compared to the ASC (0.027 OD) and SVF (0.004 OD) treatment groups. Similarly, the levels of IL-12 were higher in the sera of vehicle-treated EAE mice (0.094 OD) than the SVF-treated (0.052 OD) and ASC-treated (0.055 OD) EAE mice. Moreover, TNFα was detected at a relatively high level in the sera from the vehicle treatment group (0.048 OD) compared to the sera from the ASC treatment group (0.013 OD), and the levels SVF treatment group were undetected. Likewise, the levels of IFNγ were undetectable in the sera from SVF-treated EAE mice, whereas the IFNγ levels were highest in the ASC-treated (0.047 OD) compared to the vehicle-treated (0.006 OD) EAE mice. ASC treatment (0.070 OD) induced the highest levels of IL-6 in the sera compared to SVF (0.033 OD) and vehicle (0.022 OD) treatments. The levels of TGFβ were reduced with SVF (0.052 OD) and ASC (0.055 OD) treatment compared to the vehicle treatment group (0.094 OD; Fig. 4).

### Frequencies of splenic immune cells are enhanced following treatment with SVF cells and ASCs

**Fig. 5:**
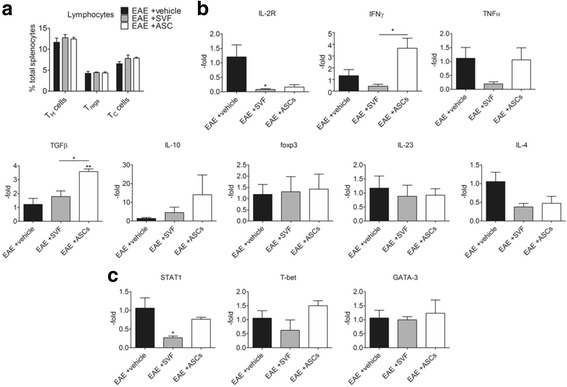
Analysis of the spleens from EAE mice 6 days following treatment with vehicle, SVF cells, or ASCs. **a** Frequencies of T cell subsets that were altered after treatment. **b** Gene expression levels of cytokines relevant to T cell differentiation into specific subsets. **c** Gene expressions of the key transcription factors relevant to the T_H_1/T_H_2 balance that were altered with treatment. For each group (*n* = 3), values were represented as the mean ± SEM. *P < 0.05; *P < 0.01; ***P < 0.001

The frequencies of T cell subsets in the spleens showed slight increases following SVF and ASC treatments. For the T_H_ cells, the SVF-treated (12.75 ± 0.79%) and ASC-treated (12.44 ± 0.41%) EAE groups had higher frequencies than the vehicle-treated EAE group (11.67 ± 0.01%). The frequency of Tregs 6 days after treatment showed 4.46 ± 0.13% and 4.36 ± 0.24% Tregs from SVF-treated and ASC-treated EAE mice, respectively, compared to the vehicle-treated EAE mice (4.25 ± 0.47% Tregs). For the T_C_ cells, ASC treatment (7.86 ± 0.25%) and SVF treatment (7.83 ± 0.79%) resulted in the higher frequencies of T_C_ cells than the vehicle treatment (6.56 ± 0.49%). The largest frequency in B cells following treatment was seen in the SVF-treated EAE mice (6.30 ± 4.71%) followed by the vehicle-treated (1.18 ± 1.09%) and ASC-treated (0.21 ± 0.06%) EAE mice (Fig. 5a).

### Splenic cytokine profile following SVF treatment promoted Tregs and diminished T_H_1, T_H_17, and T_H_2 cells

Gene expression of inflammatory cytokines and associated receptor, IL-2R, pertinent to EAE were measured from spleens 6 days following treatment with SVF cells and ASCs and normalized to those of the vehicle treatment group. The gene expression of IL-2R showed marked reductions following treatment with SVF cells (0.08 ± 0.03-fold; *P* < 0.05) and ASCs (0.17 ± 0.08-fold) compared to the vehicle treatment group (1.21 ± 0.42). The levels of IFNγ in the SVF-treated EAE mice were reduced 0.42 ± 0.17-fold (*P* < 0.05) compared to the vehicle treatment group (1.32 ± 0.51-fold), which was significantly less than the IFNγ levels of ASC-treated EAE mice (3.67 ± 0.86-fold). Similarly, the levels of TNFα were diminished in the spleens of EAE mice following treatment with SVF cells (0.20 ± 0.08-fold) compared to treatment with vehicle (1.12 ± 0.39-fold) and ASCs (1.06 ± 0.43-fold). Gene expression levels of IL-12 showed modest reductions in the SVF (0.86 ± 0.22-fold) and ASC (0.64 ± 0.11-fold) treatment groups compared to the vehicle (1.07 ± 0.29). Likewise, the gene expression levels of IL-23 did not reveal large changes following treatment with SVF cells (0.88 ± 0.39-fold), ASCs (0.92 ± 0.22-fold), and vehicle (1.17 ± 0.43-fold). The spleens from SVF-treated (0.84 ± 0.30-fold) and ASC-treated (0.82 ± 0.18-fold) EAE mice showed comparable IL-6 levels compared to the vehicle-treated EAE mice (1.03 ± 0.19-fold). The gene expression levels of IL-4 were also greatly reduced following SVF (0.38 ± 0.10-fold) and ASC (0.47 ± 0.19-fold) treatments compared to the vehicle treatment (1.05 ± 0.25-fold; Fig. 5b).

On the contrary, treatments with SVF cells and ASCs enhanced the gene expression levels of TGFβ to 1.79 ± 0.40-fold and 3.59 ± 0.19-fold higher than the vehicle treatment group (1.23 ± 0.43-fold). Higher gene expression levels of IL-10 were induced in spleens of SVF-treated (4.54 ± 2.86-fold) and ASC-treated (14.06 ± 10.58-fold) EAE mice compared to the vehicle-treated EAE mice (1.35 ± 0.55-fold). The levels of Foxp3 were 1.42 ± 0.67-fold and 1.30 ± 0.68-fold higher with ASC and SVF treatment, respectively, compared to the vehicle group (1.19 ± 0.44-fold; Fig. 5b).

To correlate the gene expression of cytokines profiled above, the gene expressions of transcription factors that regulate T_H_1 and T_H_2 cells were measured in the splenocytes of EAE mice 6 days after treatment. Quantitative comparison of STAT1 showed a marked reduction in gene expression after treatment with SVF cells (0.27 ± 0.04-fold; *P* < 0.05) as well as a reduction after ASC treatment (0.76 ± 0.05-fold) compared to the vehicle group (1.06 ± 0.27-fold). The levels of T-bet were modestly reduced with SVF treatment (0.62 ± 0.37-fold) compared to the EAE mice treated with vehicle (1.06 ± 0.26-fold) while an increase was measured following ASC treatment (1.50 ± 0.18-fold). Gene expression levels of GATA-3 were comparable in the spleens from EAE mice treated with vehicle (1.06 ± 0.27-fold), SVF cells (1.00 ± 0.11-fold), and ASCs (1.23 ± 0.47-fold; Fig. 5c).

## Discussion

Unlike the demyelinating phase at late-stage EAE disease [11], the immune system activity during the inflammatory, autoreactive phase at early disease is more robust. Initially, naïve T cells were activated and differentiated into the T_H_1 and T_H_17 cells that mediate EAE disease. As in MS, these effector T cells of the adaptive immune response propagate pro-inflammatory signals throughout the peripheral lymphoid tissues and disseminate into the CNS causing pathology [16–19]. In this study, pathologic features in the CNS were seemingly comparable amongst all the groups. Although SVF-treated EAE mice outperformed the ASC-treated EAE mice in behavioral assessments, the severity of disease initially revealed enhanced clinical features of disease directly after treatment was administered with SVF cells, and more so, ASCs. Analysis of cellular frequencies within the CNS tissues after 6 days revealed increased levels of lymphohematopoietic cells including T_H_, T_C_, and B cells following both SVF and ASC treatments. Furthermore, the microglia and macrophage populations were collectively increased in CNS tissues after ASC treatment, and the alternative activation phenotype was most enhanced compared to the classical activation phenotype. Regardless of phenotype, the known functions of macrophages and activated microglia exposed to an inflammatory milieu contribute to pathology [20–22] and, in this case, promoted demyelination following ASC treatment. These results conclusively showed that ASC treatment was unable to counter the inflammatory phase of the disease and did not provide therapeutic benefit. On the contrary, improvements that ultimately led to partial recovery of motor function in the SVF-treated EAE mice began a week following treatment and continued for the remainder of the trial.

A deeper look into the CNS showed small increases in the lymphohematopoietic cells and lymphocyte populations following SVF treatment after 6 days. At this time, treatment with SVF cells and, to a greater extent, the ASCs induced high levels of IL-6. The rapid production of IL-6 by ASCs was further demonstrated in vitro. IL-6 is a potent growth factor for T cells that induces proliferation and survival of activated T_H_ cells [23, 24], enhances the cytolytic activity of T_C_ cells [25], and modulates the T_H_1/T_H_2 balance towards T_H_2 [24, 26]. Not only is IL-6 powerful enough to protect T_H_ cells from activation-induced cell death, antigen-specific T_H_ cells expand more vigorously under IL-6 influence during immunization [24, 27]. These findings suggest that ASCs at early-stage EAE can shift the T_H_1/T_H_2 balance towards T_H_2. However, the ASC-induced secretion of IL-6 promotes untoward effects during the early, inflammatory phase of EAE when high effector T cell activities are prominent.

Although studies have demonstrated that IL-6 is a co-stimulator to T cell proliferation independent of IL-2, IL-2 is known to be the chief stimulator of T cell proliferation [24, 28]. Treatment with SVF cells potently abrogated T cell-induced pathology by thwarting IL-2 signaling. SVF markedly reduced IL-2 in circulation and diminished the gene expression of IL-2R. In doing so, the products of T_H_1, T_H_2, and T_H_17 were correspondingly reduced. In all groups, the progressive CNS damage ensued soon after induction of EAE. More importantly, early-stage treatment with SVF could rapidly overcome the pro-inflammatory signals and impede further propagation of autoreactive responses. With diminished IFNγ, TNFα, IL-4, IL-12, and IL-23, the encephalitogenic T cells were no longer actively causing CNS damage after SVF treatment. Furthermore, our data suggests that those effector cells that had infiltrated into CNS tissues did not continue to proliferate and promote inflammation.

As this disease is driven by an autoimmune response, analysis of the peripheral immune system activities, which precedes the CNS pathology, revealed novel insight into the mechanisms of SVF and ASC treatments. The data indicate that SVF treatment abrogated the differentiation and activity of T_H_1 cells to prevent further CNS damage. During disease, naïve T cells respond to inflammatory disease milieus via IFNγ and IL-12 signaling that translocates to dimerize STAT1 as an active transcription factor. STAT1 homodimerization mediates the transcription of T-bet, the master regulator of T_H_1 cell differentiation. In turn, the pro-inflammatory cytokine IFNγ is produced by the T_H_1 [29–32]. Similarly, GATA-3 is the primary transcriptional regulator of T_H_2 differentiation that results in the production of IL-4 [30, 31]. Diminished gene expression and production of IFNγ, IL-12, IL-4, STAT1, T-bet, and GATA-3 suggest treatment with SVF cells altered the differentiation and physiological function of both T_H_1 and T_H_2 cells.

Corresponding with our previous study at late-stage disease, treatments with SVF cells and ASCs were able to promote high levels of IL-10 and induction of Tregs. The induction of Tregs is necessary to counter inflammatory milieus and restore immune tolerance [33–35]. With high levels of IL-10 and TGFβ, naïve T cells are induced to express Foxp3 which is indicative of differentiation of Tregs [30, 34, 36]. In this study, we detected markedly high gene expression levels of IL-10 and TGFβ in the spleens of EAE mice after 6 days following treatment with SVF cells and ASCs. Considering the small increases in Tregs and correlative Foxp3 levels in the spleen, six days after treatment may be too soon to detect the increased production of IL-10 in the circulation. Nevertheless, SVF cells directly demonstrated the rapid anti-inflammatory potency through the marked increases in TGFβ and IL-10 gene expression when placed in the pro-inflammatory EAE disease milieu in vitro*.*

## Conclusion

**Fig. 6:**
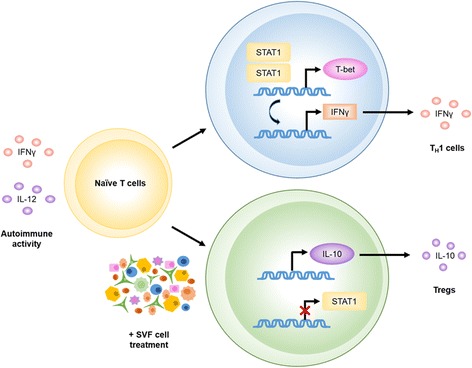
Comprehensive representation of mechanistic alterations to the pathways that lead to differentiation of T cells following SVF treatment

Together, SVF treatment at early-stage EAE provides meaningful immunomodulatory and anti-inflammatory effects resulting in partial restoration of motor function. Treatment with SVF cells could halt the function of effector T cells while promoting an environment that supports the induction of Tregs in the periphery 6 days after treatment and suggests that SVF treatment further attenuated the adaptive immune system that propagated autoimmune responses in the periphery and CNS tissues. Furthermore, we showed that during the early stage of EAE disease when T cell-mediated activities are pronounced, treatment with ASCs enhances the expansion of T cells through IL-6 signaling. Although ASCs may have also contributed to the suppression of T_H_1 cells, the confounding effects did not lead to improvements in the CNS (Fig. 6).

This evidence is the first to be reported on the therapeutic effects of SVF cells during the inflammatory phase of EAE disease. Furthermore, the potential mechanisms for SVF cells that were demonstrated in vivo and in vitro suggest modulation of T cell differentiation and function. These results suggest alterations to autoreactive T cells as well as to the cells administered as therapy. The translation implications from this study support the use of adipose stem cell-based therapeutics while highlighting the importance of monitoring disease to determine the timing of administration to prevent adverse effects.

## Abbreviations

ASCs: Adipose-derived stem cells
CCM: Complete culture media
CNS: Central nervous system
DPI: Days post induction
EAE: Experimental autoimmune encephalomyelitis
GFP: Green fluorescent protein
HBSS: Hank’s balanced salt solution
IFNγ: Interferon-γ
IL: Interleukin
IP: Intraperitoneal
MOG: Myelin oligodendrocytes glycoprotein
MS: Multiple sclerosis
PBS: Phosphate buffered saline
qPCR: Quantitative reverse transcriptase polymerase chain reaction
SVF: Stromal vascular fraction
TGFβ: Transforming growth factor-β
T_H_1: T helper type I
TNFα: Tumor necrosis factor-α
Tregs: Regulatory T cells
